# Magnetic resonance imaging of the cirrhotic liver: diagnosis of hepatocellular carcinoma and evaluation of response to treatment - Part 1

**DOI:** 10.1590/0100-3984.2015.0132

**Published:** 2017

**Authors:** Miguel Ramalho, António P. Matos, Mamdoh AlObaidy, Fernanda Velloni, Ersan Altun, Richard C. Semelka

**Affiliations:** 1 Department of Radiology, University of North Carolina at Chapel Hill, Chapel Hill, NC, USA, and Hospital Garcia de Orta, Almada, Portugal.; 2 Department of Radiology, University of North Carolina at Chapel Hill, Chapel Hill, NC, USA, and King Faisal Specialist Hospital and Research Center, Riyadh, Saudi Arabia.; 3 Department of Radiology, University of North Carolina at Chapel Hill, Chapel Hill, NC, USA.

**Keywords:** Magnetic resonance imaging, Liver Cirrhosis, Image enhancement, Contrast media

## Abstract

Magnetic resonance imaging (MRI) is the modern gold standard for the noninvasive evaluation of the cirrhotic liver. The combination of arterial phase hyperenhancement and delayed wash-out allows a definitive diagnosis of hepatocellular carcinoma (HCC) in patients with liver cirrhosis or chronic liver disease, without the requirement for confirmatory biopsy. That pattern is highly specific and has been endorsed in Western and Asian diagnostic guidelines. However, the sensitivity of the combination is relatively low for small HCCs. In this two-part review paper, we will address MRI of the cirrhotic liver. In this first part, we provide a brief background on liver cirrhosis and HCC, followed by descriptions of imaging surveillance of liver cirrhosis and the diagnostic performance of the different imaging modalities used in clinical settings. We then describe some of the requirements for the basic MRI technique, as well as the standard MRI protocol, and provide a detailed description of the appearance of various types of hepatocellular nodules encountered in the setting of the carcinogenic pathway in the cirrhotic liver, ranging from regenerative nodules to HCC.

## Abstract (pt)

A ressonância magnética (RM) é o método padrão para a avaliação não invasiva do fígado cirrótico. A combinação de hiper-realce arterial e *wash-out* tardio permite um diagnóstico definitivo de carcinoma hepatocelular (CHC) em pacientes com cirrose hepática ou doença hepática crônica, sem a necessidade de biópsia confirmatória. Este padrão é altamente específico e tem sido utilizado por *guidelines* de diagnóstico ocidentais e asiáticas. No entanto, a sensibilidade desta combinação é relativamente baixa para CHCs pequenos. Neste artigo de revisão de duas partes, irá ser efetuada uma revisão do papel da RM na avaliação do fígado cirrótico. Na primeira parte, faremos uma breve revisão sobre cirrose hepática e CHC, seguido da vigilância da cirrose hepática por imagem e desempenho diagnóstico das diferentes modalidades de imagem utilizadas na prática clínica. Depois, descreveremos alguns dos requisitos técnicos básicos para RM, protocolos de RM e uma descrição detalhada do aparecimento dos diferentes nódulos hepatocelulares encontrados no contexto da via carcinogênica do fígado cirrótico, desde nódulos regenerativos a CHC.

## INTRODUCTION

Hepatocellular carcinoma (HCC) is the sixth most commonly diagnosed cancer worldwide^(1)^, developing within a cirrhotic context in up to 90% of cases^(2)^. Regardless of the underlying cause, cirrhosis is the single most important risk factor for HCC. The 5-year cumulative incidence of HCC has been shown to be 8-30% in patients with cirrhosis^(3)^. Conversely, the annual incidence of HCC is < 0.5% in patients without cirrhosis^(3)^.

Cirrhosis is a late stage of scarring (fibrosis) of the liver resulting from chronic hepatic inflammation caused by many forms of liver diseases and conditions, such as hepatitis and chronic alcohol abuse, and is characterized by the normal hepatic architecture being replaced with a mixture of parenchymal nodules and fibrosis. The magnetic resonance imaging (MRI) findings of cirrhosis reproduce those histological changes and include altered hepatic morphology, fibrosis, and cirrhotic nodules^(4)^. The spectrum of cirrhotic nodules includes regenerative nodules, low-grade dysplastic nodules, high-grade dysplastic nodules, and neoplastic nodules.

The development of HCC in a cirrhotic liver is described either as *de novo* hepatocarcinogenesis or as a multistep progression, from low-grade dysplastic nodule to high-grade dysplastic nodule, then to high-grade dysplastic nodule with microscopic foci of HCC, then to small HCC, and finally to invasive carcinoma. *De novo* hepatocarcinogenesis is presumed to occur as an alternative pathway; however, even in such cases, later progression to overt HCC takes place in a multistep fashion.

HCC may be very aggressive and frequently presents as a rapidly growing tumor, usually associated with poor prognosis and outcome, with a 5-year survival rate of less than 10%^(5)^. However, patients diagnosed in the early stages are eligible for potentially curative therapies, including surgical resection, liver transplantation, and thermal ablation treatment, such as radiofrequency or microwave ablation^(6)^. In this population, stage-driven treatment results in 5-year survival rates in the range of 50-70%^(7)^. Hence, diagnosing HCC in the early stages is critical.

## SURVEILLANCE FOR HCC

The way that surveillance for HCC is performed remains a controversial topic. Currently, ultrasound (US) is used for the surveillance of HCCs in high-risk individuals. Gray-scale US has been the modality most commonly used for screening or surveillance for HCC. A recently updated practice guideline for the management of HCC by the American Association for the Study of Liver Diseases (AASLD) recommended that surveillance of HCC be centered on US at 6-month intervals^(8)^, because alpha-fetoprotein determination lacks sensitivity and specificity for effective surveillance, which appeared to justify the omission of alpha-fetoprotein testing from those new recommendations for HCC surveillance. However, some authors argue that alpha-fetoprotein testing is still useful and should be regarded as complementary to US for the surveillance of HCC, various studies having shown that the sensitivity and specificity for the detection of HCC improve considerably when the two tests are used in combination^(9,10)^.

The fact that US is the most common initial imaging test used for the screening and surveillance of HCC is primarily due to its ease of access, absence of risks, non-invasiveness, good acceptance by patients, and relative lower initial per-study cost compared with computed tomography (CT) and MRI. According to the updated AASLD and European Association for the Study of the Liver (EASL) guidelines, the HCC diagnostic algorithm starts from suspected nodules found on US surveillance. The reported sensitivity and specificity are variable, ranging from 33% to 96%^(11)^, and are highly dependent on the expertise of the operator, the morphotype of the patient, and the quality of the equipment. Previous studies have shown that the HCC detection rate of US is significantly lower than is that of multidetector CT and MRI^(12)^. In addition, US is ineffective in detecting small HCCs.

**Figure 1:**
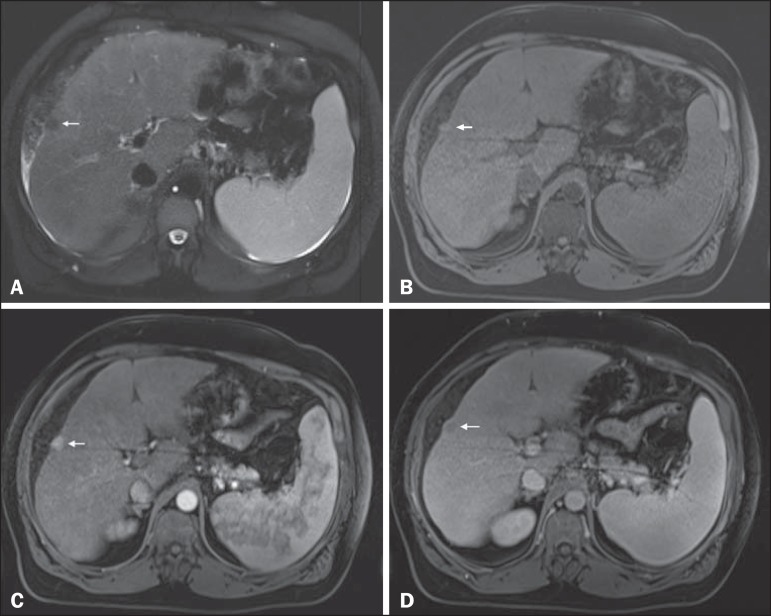
*Small* HCC. Axial SSFSE T2-weighted image, with fat suppression (**A**), axial pre-contrast 3D-GRE T1-weighted image, with fat suppression (**B**), and axial postcontrast 3D-GRE T1-weighted images, with fat suppression, in the arterial and interstitial phases (**C** and **D**, respectively). A 10-mm subcapsular nodule is depicted with minimal high signal intensity on the T2-weighted image (arrow, **A**), low signal intensity on a T1-weighted image (arrow, **B**), arterial hyperenhancement (arrow, **C**) and wash-out on the delayed phase (arrow, **D**), consistent with HCC.

According to the AASLD guidelines, when a nodule with a diameter < 10 mm is detected by US, it should be followed by US every 3 months until the nodule is no longer visualized, remains stable for 18-24 months, or grows to ≥ 10 mm in size (Figure 1), at which point MRI or CT is recommended. To date, the confirmation of HCC has been based on the hemodynamic feature of the nodules (i.e., enhancement in the arterial phase and wash-out in the portal or equilibrium phase.

MRI offers several advantages over CT, including lack of ionizing radiation, superior contrast resolution, a variety of sequences with their different image weighting, and a higher safety profile of contrast agents, together with the ability to use extracellular and hepatocyte-specific contrast media, to provide a detailed evaluation of the biliary tree, and to characterize the occasional problematic pseudo-lesions identified on US or CT, such as focal fatty infiltration and focal fatty sparing, as well as showing better overall accuracy in the detection and characterization of focal lesions^(13)^. However, MRI may have some disadvantages, because it requires better patient cooperation for single breath-hold liver imaging and longer overall scanning time, as well as because it may be contraindicated in patients with certain metal implants, claustrophobia, or pacemakers.

Previous studies have suggested that CT has a lower sensitivity for detecting dysplastic nodules, small HCCs, and diffuse HCCs than does MRI^(11,14)^. In addition, several studies have demonstrated that the sensitivity and specificity of dynamic MRI is greater than is that of dynamic CT for the detection and characterization of HCC of all sizes, reporting sensitivities of 61-90% for MRI, compared with 52-78% for CT^(15-18)^. An optimized, dynamic T1-weighted gradientrecalled echo (GRE) with individually tailored arterial phase timing has shown high sensitivity and specificity (> 95%)^(19)^. The sensitivity of MRI varies with tumor size, although it has been estimated to be nearly 100% in HCCs larger than 20 mm. The detection of smaller tumors remains challenging, and MRI continues to outperform CT in this area, with a reported sensitivity for the detection of HCCs measuring 10-20 mm of 84%, compared with 47% for MRI and for CT^(15)^. Concerning HCCs measuring < 10 mm, a recent meta-analysis showed that both techniques showed quite low sensitivity, although the sensitivity was lower for CT than for MRI (31% vs. 48%)^(20)^. For HCCs measuring < 10 mm, the estimated per-lesion sensitivity is relatively higher for MRI than for CT and may be further increased with the use of hepatobiliary contrast agents, particularly gadoxetic acid^(20)^.

Because of its higher diagnostic accuracy for the detection and characterization of HCCs, together with technical advancements ensuring superior and more reproducible image quality, MRI has gained an increasingly central role in evaluating patients with chronic liver disease. Many physicians, including liver specialists and radiologists, currently prefer dynamic contrast-enhanced MRI to CT for the evaluation of liver nodules ≥ 10 mm in size. Although many MRI features deserve attention, enhancement is still considered the most important. Despite numerous technological developments and improvements in recent years, the proof of HCC is still based on the hemodynamic feature of the nodules (i.e., enhancement in the arterial phase and wash-out in the portal or equilibrium phase). Using hemodynamic criteria alone has limitations, because small HCCs frequently show an atypical enhancement pattern. A study conducted by Forner et al.^(21)^ suggested that current CT and MRI criteria are highly specific but can be insufficiently sensitive for diagnosing HCCs, given that 30-40% of patients with cirrhosis and HCC may not meet the typical enhancement criteria of arterial enhancement and venous wash-out. This is more common for lesions < 20 mm in size, which often show discrepant enhancement patterns.

## MRI TECHNICAL REQUIREMENTS AND PROTOCOL

An in-depth discussion of liver MRI techniques is beyond the scope of this review. However, there are a few points worth noting regarding the MRI technique.

To diagnose HCC with MRI, specific minimum technical requirements^(22)^, as outlined in Table 1, must be met in order to reduce the number of biopsies or repeat MRI studies. In addition, MRI protocols for HCC surveillance should be standardized in order to allow repeatability and consistency.

**Table 1:** Minimum technical specifications for MRI of HCC, as outlined by the Organ Procurement and Transplantation Network.

Feature	Specification
Field strength	1.5 T or greater
Coil type	Phased-array multichannel torso coil
Minimum sequences	Pre-contrast and dynamic gadolinium-enhanced T1-weighted GRE (3D preferable)
	T2-weighted (with and without fat suppression)
	In-phase and out-of-phase T1-weighted
Injector	Dual-chamber power injector
Contrast agent injection rate	For extracellular gadolinium chelate without dominant biliary excretion, 2–3 mL/s
Mandatory phases on contrast-enhanced MRI	Pre-contrast T1-weighted, late arterial phase, portal venous phase, delayed phase
Dynamic phase timing	Use of bolus-tracking method for timing contrast arrival for late arterial phase imaging is preferable; portal venous phase (35–55 s after initiation of late arterial phase imaging); delayed phase (120–180 s after initial contrast injection)
Slice thickness	For dynamic studies, 5 mm or less; for other imaging studies, 8 mm or less
Breath holding	Maximum length of series requiring a breath-hold, which should be approximately 20 s, with a minimum matrix of 128 × 256

The field strength should be 1.5 T or greater. A standard protocol is based on dynamic fat-suppressed post-contrast T1-weighted 3D-GRE sequences, combined with in-phase and out-of-phase T1-weighted GRE sequences (chemical shift imaging), as well as T2-weighted sequences with and without fat suppression. T1-weighted sequences are acquired in a breath-hold that should be less than 20 s in order to reduce the risk of respiratory motion artifacts. T2-weighted images are usually acquired with single-shot fast spin-echo (SSFSE) technique due to its robustness to motion but may also be acquired during breath-hold or in a free-breathing fashion with respiratory or diaphragmatic motion gating. For dynamic studies, the section thickness should be 5 mm or less, whereas it should be 8 mm or less for all other studies.

Contrast injection should be performed with a dual-chamber power injector at a rate of 2-3 mL/s. Dynamic imaging should include the late arterial, portal venous, and delayed phases. An optimal late arterial phase is recognized when contrast is present in the portal veins and absent in the hepatic veins. That phase is critically important in maximizing visualization of arterial phase hyperenhancement. However, gadolinium-based contrast agent perfusion of tumors with arterial vascularization is a transient phenomenon, and a mistiming of only a few seconds during the arterial phase image acquisition may render the exam less diagnostic for HCC detection^(14)^. Several techniques, including test bolus, fluoroscopic triggering, and bolus tracking with or without automatic bolus detection, can help optimize the timing of image acquisition^(23)^.

This represents the minimum technical specifications for MRI in HCC screening/evaluation. Although additional sequences may be added to the protocol (see Part 2 of this review), they remain optional.

## FEATURES OF CIRRHOTIC NODULES

The diagnosis of HCC by CT or MRI is predominantly centered on sequential changes in the intra-nodular blood supply during hepatocarcinogenesis. Regenerative nodules show a blood supply comparable to that of background liver tissue, borderline lesions such as dysplastic nodules or early HCCs show wide variations in blood supply, and advanced HCCs are predominantly supplied by anomalous arteries. Development of a hepatic arterial supply may be associated with a higher grade of dysplasia, in which unpaired arterioles begin to be prominent. Through the accumulation of cytological alterations, neoangiogenesis, and the gradual decrease in the expression of certain organic anionic transporting polypeptides, these lesions progressively dedifferentiate, leading to the development of HCC. The imaging diagnosis of HCC is primarily based on sequential changes in the intra-nodular blood supply during hepatocarcinogenesis. In the classical multistep hepatocarcinogenesis pathway concept, as cellular atypia progresses toward malignancy, the normal portal venous supply is slowly lost, being replaced by that coming from numerous small unpaired arteries formed via neoangiogenesis. Consequently, many high-grade dysplastic nodules exhibit altered enhancement patterns, including isolated hyperenhancement in the arterial phase fading to isointensity in the venous and delayed phases. This can occur at an intermediate stage when a nodule loses portal vascularization but does not gain substantial arterial vascularization. In addition, borderline lesions such as high-grade dysplastic nodules or early HCCs can show wide variation in blood supply. That is reflected in recent publications in which many high-grade dysplastic nodules and early HCCs were found to be isointense or hypointense in the arterial phase^(24,25)^.

### Regenerative nodules

**Figure 2:**
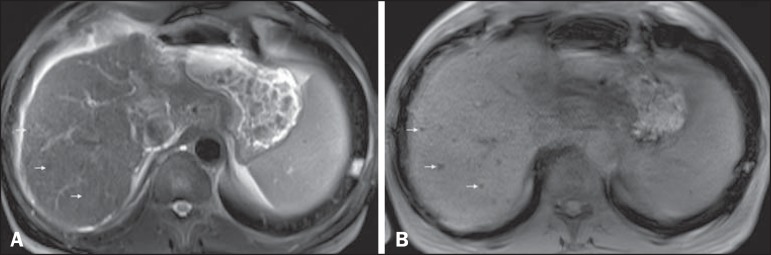
*Siderotic nodules*. Axial SSFSE T2-weighted image, with fat suppression (**A**) and axial in-phase 3D-GRE T1-weighted image (**B**). Multiple siderotic nodules, showing low signal intensity on T2- and T1-weighted images, can be seen throughout the hepatic parenchyma. Note that the low signal intensity of the iron-containing nodules is better depicted on the T1-weighted scans with longer echo times.

Regenerative nodules are benign and consist of proliferating normal liver cells surrounded by a fibrous stroma, formed during the normal response to a wide variety of liver injuries or altered circulation^(26)^. In regenerative nodules, the cells are histologically normal and lack clonal features. Because of their histopathological nature, regenerative nodules are often indistinct on T1- and T2-weighted images. However, they may present higher T1 signal intensity compared with background liver tissue, which may be due to the presence of metal-binding proteins, proteins *per se*, or, infrequently, lipids^(27)^. Out-of-phase images are helpful in characterizing nodules with high T1 signal intensity, such as fatty nodules. When out-of-phase and in-phase images are compared, the presence of a small amount of fat results in signal loss because the signals from fat and water cancel each other out. Making that separation is important because large (> 15 mm) fatty nodules (hyperintense on in-phase T1-weighted images with a loss of signal on out-of-phase T1-weighted images) strongly suggest malignancy. In addition, Sano et al.^(25)^ showed that up to 40% of small (≤ 20 mm) early HCCs contain intracytoplasmic fat. Conversely, the presence of numerous fatty nodules (steatotic nodules) < 10 mm suggests benignity^(28)^. Regenerative nodules occasionally accumulate iron (siderotic nodules), in which case they will show low signal intensity on all MRI sequences, due to susceptibility effects (Figure 2).

Regenerative nodules have dominant portal vascularization; therefore, the enhancement is comparable to background liver enhancement throughout all phases of dynamic evaluation. It is worth mentioning that post-processed subtraction images are often helpful and appear to be more accurate than are mere qualitative evaluations or simple region-of-interest signal measurements. Subtraction imaging may allow accurate detection of arterial enhancement in hepatic nodules, which appear mildly hyperintense on pre-contrast T1-weighted images^(29)^.

### Dysplastic nodules

Dysplastic nodules are defined as regenerative nodules containing atypical cells with nuclear crowding and architectural derangement, together with a variable number of unpaired arterioles or capillaries without definite histological signs of malignancy.

**Figure 3:**
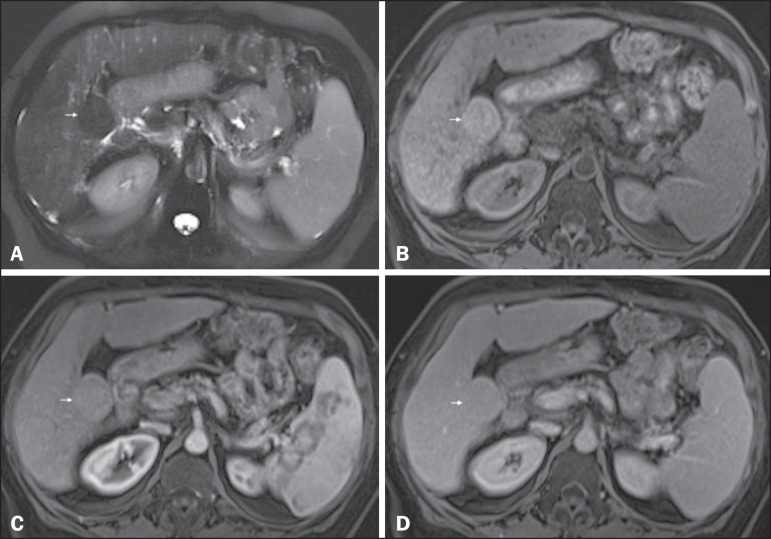
*Low-grade dysplastic nodule in a patient with chronic hepatitis C virus infection*. Axial SSFSE T2-weighted image, with fat suppression (**A**), axial pre-contrast 3DGRE T1-weighted image, with fat suppression (**B**), and axial post-contrast 3D-GRE T1-weighted images, with fat suppression, in the arterial and interstitial phases (**C** and **D**, respectively). A 3.5-cm nodule is depicted with low-signal intensity on T2-weighted images (arrow, **A**) and mild high-signal intensity on pre-contrast T1-weighted image (arrow, **B**). On the dynamic post-contrast images, the lesion shows the same pattern of enhancement as the background liver parenchyma (arrow, **C** and **D**). Although unusual, this nodule was prospectively considered a large regenerative nodule or a lowgrade dysplastic nodule. The histopathological correlation was consistent with a lowgrade dysplastic nodule.

In imaging studies, dysplastic nodules are seen in 15- 20% of cirrhotic livers, although they occur often more commonly in pathologic specimens^(30)^. Dysplastic nodules are histologically classified as either low-grade or high-grade, depending on the level of cellular and structural atypia^(31)^. On T1-weighted images, dysplastic nodules mostly show isointensity in relation to the background liver tissue, although hyperintensity is also possible as described above for regenerative nodules^(32)^. On T2-weighted images, dysplastic nodules usually show intermediate to low signal intensity, whereas early HCC is typically isointense or mildly hyperintense^(33)^. Low-grade dysplastic nodules primarily display enhancement characteristics similar to those of the background liver parenchyma in all dynamic phases, because they remain mainly supplied by the portal circulation (Figure 3). Although low-grade dysplastic nodules cannot be differentiated from regenerative nodules, that information does not have much clinical relevance. Low-grade dysplastic nodules are considered premalignant lesions, despite their low malignant potential. In contrast, high-grade dysplastic nodules have high malignant potential, being recognized as a precursor of HCC. High-grade dysplastic nodules are histologically similar to well-differentiated HCCs^(34)^. High-grade dysplastic nodules progress to HCC at up to 46%/year^(31,35)^. Therefore, identification of high-grade dysplastic nodules has significant prognostic repercussions.

As cellular atypia progresses toward malignancy, the blood supply becomes more arterialized. Nevertheless, as mentioned above, the portal and arterial supply to high-grade dysplastic nodules is variable and inconsistent. On MRI, high-grade dysplastic nodules demonstrate variable signal intensity on T1-weighted images, depending on their content, whereas they are usually isointense or hypointense on T2-weighted images. Most high-grade dysplastic nodules hypovascular^(36)^, although they may exhibit arterial enhancement similar to that seen in HCC^(37)^, despite fading to isointensity in the later phases^(38)^, without wash-out, because the supply from the portal venous system remains comparable to that observed for the background liver tissue. Establishing the differential diagnosis between high-grade dysplastic nodules and early HCC on the basis of imaging and pathological characteristics may be difficult.

**Figure 4:**
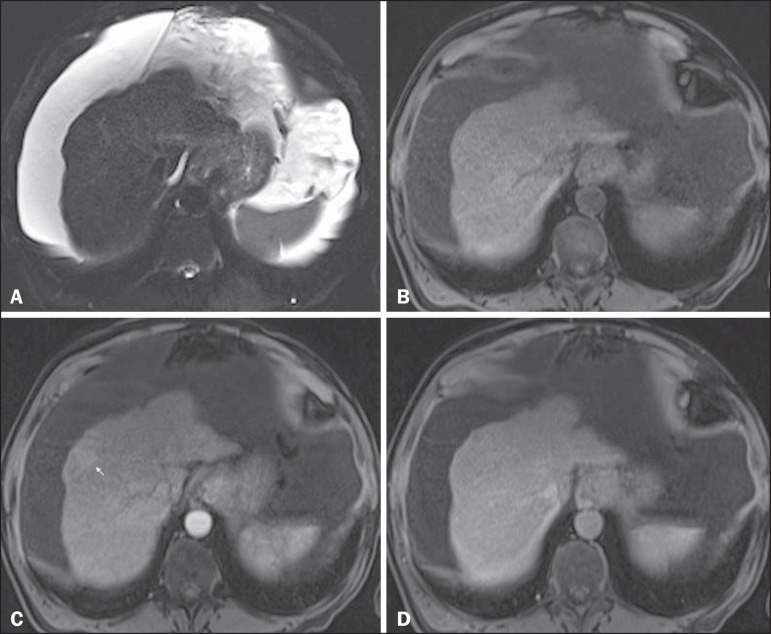
*High-grade dysplastic nodule* vs. *perfusion abnormality*. Axial FSE T2-weighted image, with fat suppression (**A**), axial pre-contrast 3D-GRE T1-weighted image, with fat suppression (**B**), and axial post-contrast 3D-GRE T1-weighted images, with fat suppression, in the arterial and interstitial phases (**C** and **D**, respectively). In the context of a patient with cirrhosis, one hepatic nodule is depicted only on the arterial phase (arrow, **c**), showing hypervascularity with no wash-out on the delayed phase (**D**). This nodule is not well depicted in the pre-contrast images, due to the isointense signal, comparable to that of the background liver parenchyma, on T1- and T2-weighted images (**B** and **A**, respectively). This abnormality is peripheral, not well-defined, and seen only in the arterial phase, raising the suspicion of perfusion abnormality. The differential diagnosis includes high-grade dysplastic nodule and this abnormality should therefor be followed closely, preferentially with hepatobiliary contrast-enhanced scans.

**Figure 5:**
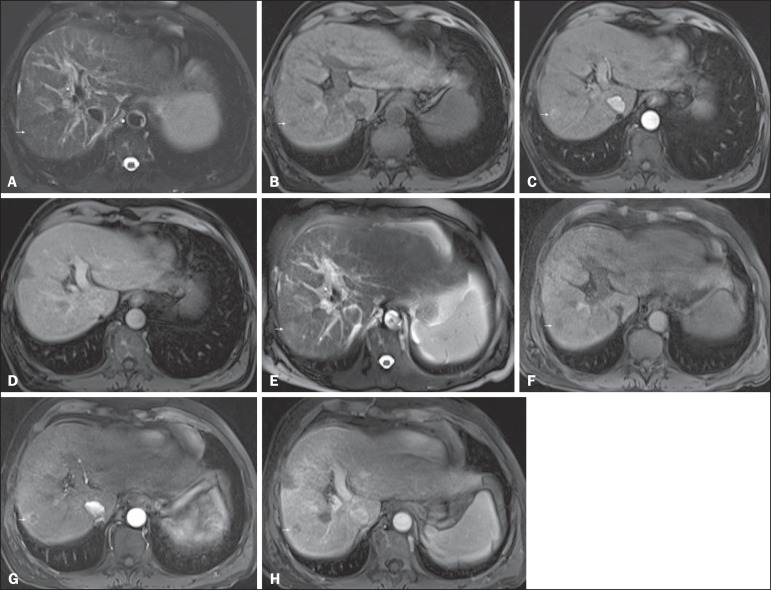
*Progression from high-grade dysplastic nodule to HCC in a patient with a history of chemoembolization for HCC*. Axial SSFSE T2-weighted image, with fat suppression (**A**), axial pre-contrast 3D-GRE T1-weighted image, with fat suppression (**B**), and axial post-contrast 3D-GRE T1-weighted images, with fat suppression, in the arterial and interstitial phases (**C** and **D**, respectively). In the right hepatic lobe, a small high-grade dysplastic nodule is depicted, showing an isointense signal on the T2-weighted image (arrow, **A**), mild low signal intensity on a T1-weighted image (arrow, **B**), slight arterial hyperenhancement (arrow, **C**) and no perceived wash-out in the delayed phase (**D**). Axial SSFSE T2-weighted image, with fat suppression (**E**), pre-contrast 3D-GRE T1-weighted images, with fat suppression (**F**), and post-contrast 3D-GRE T1-weighted images, with fat suppression, in the arterial and interstitial phases (**G** and **H**, respectively) in the same patient 2 months later. The follow-up MRI shows the same nodule with mildly high signal intensity on the T2-weighted image (arrow, **E**), peripherally arterial hyperenhancement (arrow, **G**) and clear wash-out in the late phase (arrow, **H**). This case illustrates the importance of short follow-up studies for arterially enhanced nodules without typical characteristics of HCC.

**Figure 6:**
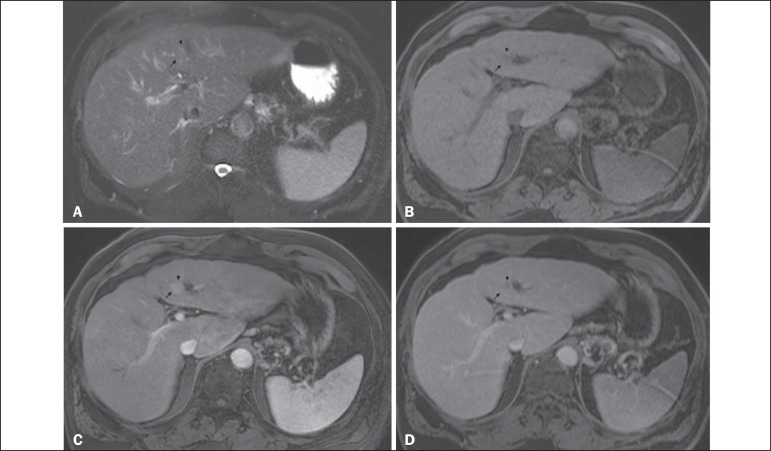
*Small HCC arising from a high-grade dysplastic nodule - nodule-within-a-nodule appearance*. Axial SSFSE T2-weighted image, with fat suppression (**A**), axial pre-contrast 3D-GRE T1-weighted image, with fat suppression (**B**), and axial post-contrast 3D-GRE T1-weighted images, with fat suppression, in the arterial and interstitial phases (**C** and **D**, respectively). A heterogeneous 15-mm nodule is depicted in the left hepatic lobe (arrows, **A-D**). Within the lesion (medial aspect), there can be seen a small nodule (< 10 mm) with mildly high signal intensity on the T2-weighted image (small arrow, **A**) and low signal intensity on the pre-contrast T1-weighted image (small arrow, **B**), with hyperenhancement in the arterial phase (small arrow, **C**) and wash-out in the interstitial phase (small arrow, **D**). The remaining part of the main nodule (lateral aspect; 15 mm) shows signal intensity similar to that of the background liver parenchyma on T2- and T1-weighted images (arrows, **A** and **B**, respectively), hyperenhancement in the arterial phase (arrow, **C**), and no delayed wash-out, features consistent with a high-grade dysplastic nodule or early HCC (arrow, **D**). These features are also consistent with a small HCC arising within a high-grade dysplastic nodule (small arrow, **A**), as well as with early HCC (arrows, **A-D**), giving the lesion a typical and highly specific nodule-within-a-nodule appearance.

The presence of a small (10-20 mm) round nodule that shows increased arterial enhancement without delayed wash-out or elevated T2 signal is considered a probable high-grade dysplastic nodule. Unfortunately, those features might also be seen in perfusion abnormalities (Figure 4). Such abnormalities, also known as arterioportal shunts, are sometimes easily distinguished from high-grade dysplastic nodules by their subcapsular location and wedge- or comma-shaped configuration. However, they can be the main mimickers of high-grade dysplastic nodules, posing as a potential differential diagnosis when they are round or oval in shape. Hence, these nodules should be reevaluated every 3-6 months, preferentially using hepatobiliary contrast agents^(39)^. The establishment of an HCC within a dysplastic nodule is typically seen as an increase in size and the development of wash-out on delayed imaging (Figure 5), allowing a definitive diagnosis of HCC to be made. Less frequently, a small HCC may have a nodule-within-a-nodule appearance, if a focus of HCC originates within a high-grade dysplastic nodule (Figure 6). It is of note that high-grade dysplastic nodules and early HCCs are recognized as lesions in the "gray zone", because, when extracellular gadolinium-based contrast agents are used, they may present a broad range of vascularity and tend to display no wash-out in the later phases, which impedes the diagnosis^(21)^.

## HCC

**Figure 7:**
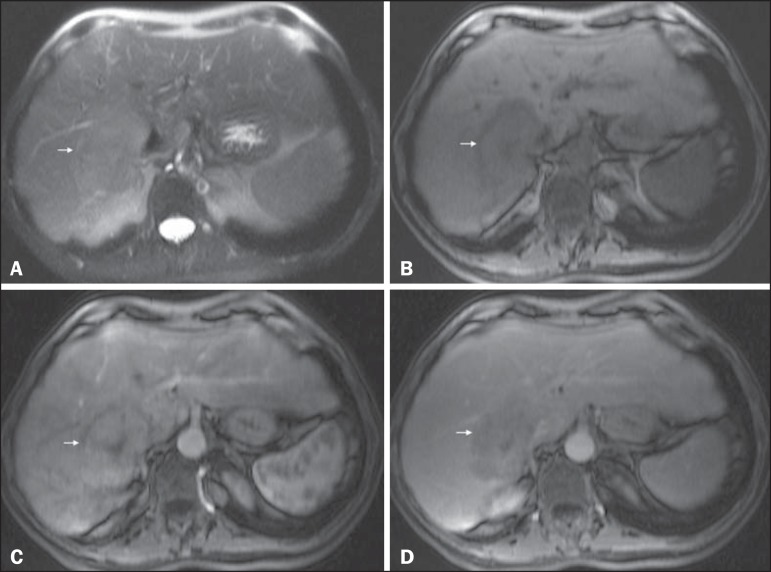
*Typical HCC in a patient with chronic hepatitis C*. Axial SSFSE T2-weighted image, with fat suppression (**A**), axial pre-contrast in-phase 3D-GRE T1-weighted image, with fat suppression (**B**), and axial post-contrast in-phase 3DGRE T1-weighted images, with fat suppression, in the arterial and interstitial phases (**C** and **D**, respectively). A 6-cm nodule is depicted in the right hepatic lobe (arrow, **A**), showing mild high signal intensity on T2-weighted image (**A**) and low-signal intensity on pre-contrast T1-weighted image (**B**). On the dynamic post-contrast images, the lesion shows arterial hyperenhancement (**C**) and delayed wash-out with pseudocapsule enhancement (**D**).

The AASLD and EASL have validated imaging criteria for the diagnosis of HCC in cirrhotic patients including arterial hyperenhancement and delayed wash-out (Figure 7).

HCCs may show a variety of MRI features, reflecting the variable architecture, grading, stromal components, and intracellular content of the tumor. Arterial-phase hyperenhancement relative to the background liver parenchyma—attributed to a shift in tumor supply from predominately from the portal vein to predominately from small arterial branches recruited during neoangiogenesis^(40)^—is the single most critical imaging feature of HCC and has a reported sensitivity of 82-93% for identifying HCC^(21,26,36,41-44)^. However, such hyperenhancement can be also seen in high-grade dysplastic nodules and arterioportal shunts^(45)^.

**Figure 8:**
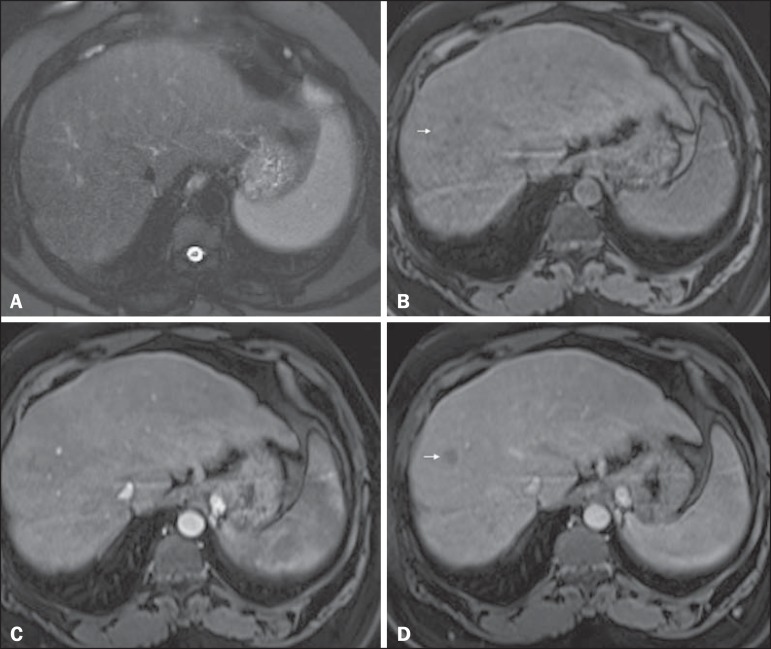
*Isovascular HCC in a patient with chronic hepatitis C*. Axial SSFSE T2-weighted image, with fat suppression (**A**), axial pre-contrast 3D-GRE T1-weighted image, with fat suppression (**B**), axial post-contrast 3D-GRE T1-weighted images, with fat suppression, in the arterial and interstitial phases (**C** and **D**, respectively). One small nodule is depicted in the right hepatic lobe (arrows, **A-D**). The nodule shows an isointense signal on T2-weighted images (**A**), and minimally high signal intensity on the pre-contrast T1-weighted image (arrow, **B**). On the dynamic postcontrast images, this lesion shows isovascular properties in the arterial phase (**C**), together with delayed washout and partial pseudocapsule enhancement (**D**). These characteristics are consistent with an isovascular HCC. The patient was treated with thermal ablation.

The superior contrast resolution and greater sensitivity to intravenous contrast media may account for the higher sensitivity of MRI relative to CT to detect hypervascular liver nodules. The combination of diffuse arterial hyperenhancement and delayed wash-out are highly specific for HCC. Delayed wash-out has been attributed in part to reduced portal venous supply to the tumor compared with the surrounding parenchyma^(40,46)^. The wash-out feature remains a subjective observation^(46,47)^ that has high sensitivity and specificity for HCC, particularly in tumors greater than 20 mm^(26,43,48-52)^. The sensitivity decreases for HCCs smaller than 20 mm^(42,43,50,51)^. This is not due to hypovascularization, an uncommon finding in HCCs (Figure 8), but rather to hypervascularization, a situation in which the HCC shows no wash-out on delayed images^(21,45,53,54)^. In one study of 60 HCCs smaller than 20 mm, hypervascularization was observed in 85%, only 61.7% of which showed wash-out^(21)^. Similarly, in another study, 51 of 131 HCCs showed arterial hyperenhancement without clear wash-out on delayed images^(54)^.

## CONCLUSION

MRI is the modern gold standard for the noninvasive evaluation of the cirrhotic liver. The combination of arterial phase hyperenhancement and delayed wash-out allows a definitive diagnosis of HCC to be made for nodules ≥ 10 mm in patients with cirrhosis or chronic liver disease, without the requirement for confirmatory biopsy.
